# Intraspecific independent evolution of floral spur length in response to local flower visitor size in Japanese *Aquilegia* in different mountain regions

**DOI:** 10.1002/ece3.8668

**Published:** 2022-03-01

**Authors:** Tsubasa Toji, Shun K. Hirota, Natsumi Ishimoto, Yoshihisa Suyama, Takao Itino

**Affiliations:** ^1^ Graduate School of Medicine, Science and Technology Shinshu University Matsumoto Japan; ^2^ 13101 Field Science Center, Graduate School of Agricultural Science Tohoku University Osaki Japan; ^3^ Faculty of Science Shinshu University Matsumoto Japan; ^4^ Department of Biology and Institute of Mountain Science Shinshu University Matsumoto Japan

**Keywords:** bumblebees, flower size, independent evolution, MIG‐seq, pollination, trait matching

## Abstract

Geographic differences in floral traits may reflect geographic differences in effective pollinator assemblages. Independent local adaptation to pollinator assemblages in multiple regions would be expected to cause parallel floral trait evolution, although sufficient evidence for this is still lacking. Knowing the intraspecific evolutionary history of floral traits will reveal events that occur in the early stages of trait diversification. In this study, we investigated the relationship between flower spur length and pollinator size in 16 populations of *Aquilegia buergeriana* var. *buergeriana* distributed in four mountain regions in the Japanese Alps. We also examined the genetic relationship between yellow‐ and red‐flowered individuals, to see if color differences caused genetic differentiation by pollinator isolation. Genetic relationships among 16 populations were analyzed based on genome‐wide single‐nucleotide polymorphisms. Even among populations within the same mountain region, pollinator size varied widely, and the average spur length of *A. buergeriana* var. *buergeriana* in each population was strongly related to the average visitor size of that population. Genetic relatedness between populations was not related to the similarity of spur length between populations; rather, it was related to the geographic proximity of populations in each mountain region. Our results indicate that spur length in each population evolved independently of the population genetic structure but in parallel in response to local flower visitor size in different mountain regions. Further, yellow‐ and red‐flowered individuals of *A. buergeriana* var. *buergeriana* were not genetically differentiated. Unlike other *Aquilegia* species in Europe and America visited by hummingbirds and hawkmoths, the Japanese *Aquilegia* species is consistently visited by bumblebees. As a result, genetic isolation by flower color may not have occurred.

## Graphical abstract

In this study, we demonstrate that long spurs in *Aquilegia buergeriana* var. *buergeriana* evolved in parallel in different mountain regions as a convergent adaptation to locally abundant large bumblebees.

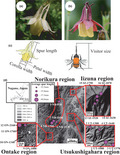

## 1 INTRODUCTION

Pollination mutualism is one of the major interaction systems between plants and animals, and through this interaction, flower visitors contribute to the reproduction of plants in different ways (Dohzono & Suzuki, 2010; Galen, 1996; Gómez et al., 2009; Herrera et al., 2006; Inoue et al., 2007; Nattero et al., 2011; Scobell & Scott, 2002). Adaptation to locally different pollinator assemblages within the distribution range of a plant species leads to local morphological specialization, which may cause trait diversification and speciation in the plants (Galen & Newport, 1987; Grant & Grant, 1965; Stebbins, 1970). Geographic variation in floral traits such as flower size and shape (Gómez et al., 2006; Nagano et al., 2014), corolla tube size (Fenster et al., 2004; Hodges, 1997), odor (Majetic et al., 2009; Pellmyr, 1986), and color (Campbell et al., 1997; Newman et al., 2012) are considered to have evolved as a local adaptation to regional pollinators. In particular, morphological matching between floral spur length and pollinator proboscis length is well known, with the textbook example being Darwin's hawkmoth and orchid (Darwin, 1877; Nilsson, 1988). In fact, geographic correlations between floral size and pollinator size have been reported in a variety of plant taxa (Alexandersson & Johnson, 2002; Anderson & Johnson, 2008; Boberg et al., 2014; Herrera et al., 2006; Johnson & Anderson, 2010; Kuriya et al., 2015; Nagano et al., 2014).

Local adaptation of floral traits to pollinators may have occurred across multiple regions, but there is little evidence as to whether variation in floral traits has occurred independently among regional populations (but see Anderson et al., 2014; Toji et al., 2021). As in textbook examples of ecological speciation (Nosil, 2012), one useful approach to understand the interaction between trait diversification and speciation in angiosperms is to combine a field analysis of local plant evolutionary adaptations with a population genetic analysis that examines genetic relationships among populations. This approach will allow us to unravel the historical patterns of floral trait evolution. Local adaptation of floral traits to pollinators may lead to speciation via the establishment of prezygotic reproductive isolation (Grant‐Stebbins model; Grant & Grant; Anderson et al., 2014; Johnson & Anderson, 2010; Stebbins, 1970) because one possible result of specialization of a trait to a particular pollinator is a lack of pollinator sharing among plant populations (Anderson & Johnson, 2008; Herrera et al., 2006; Newman et al., 2015). About 25% of plant diversification events may be associated with pollinator shifts (Van der Niet & Johnson, 2012); thus, combined analyses of local adaptation of floral traits and population genetics can shed light on the mechanisms of plant diversity (Thompson, 2005; Thompson et al., 2013). Furthermore, regional pollinator difference is the topic that is closely related to speciation and morphological diversification, yet as mentioned above, we have very limited knowledge about historical patterns of floral trait evolution. In this study, we hypothesized that the *Aquilegia* plants in each mountain region have their respective common ancestor, which would be confirmed by population genetic analysis, and their flower size independently evolved in each population as an adaptation to the local pollinator size. The purpose of this study is to provide a further perspective to understand convergent evolution related to regional pollinator differences.

In genus *Aquilegia* (Ranunculaceae), adaptive radiation to different pollinators (bumblebees, hummingbirds, and hawkmoths) has occurred. Mainly, flower color, spur length, flower orientation, and pistil length have evolved to adapt to each pollinator (Fulton & Hodges, 1999; Hodges et al., 2004). Moreover, molecular phylogenetic evidence also indicates that pollinator shifts have led to morphological diversification and speciation within this genus (Whittall & Hodges, 2007). According to Whittall and Hodges (2007), a more ancestral floral state of *Aquilegia* is purple, downward facing, short‐spurred flowers, which are pollinated by bumblebees. From plants with this floral state, taxa with red, downward facing flowers with protruding stamens and intermediate length spurs, which are pollinated by hummingbirds, were derived. Then, taxa with white and yellow long‐spurred, lateral and upward facing flowers, which are pollinated by hawkmoths, were derived from those taxa. Their results reveal an interesting pattern of species‐level diversification as a consequence of pollinator shifts, although evidence for flower trait diversification at the earlier stages of speciation is lacking. *Aquilegia* is a great model for pollinator‐driven speciation, but to observe early stages of speciation, it is useful to investigate the pattern of evolutionary morphological diversification within a single species (Sobel & Streisfeld, 2015).

In this study, we focused on evolutionary processes leading to spur length and flower color differentiation in *Aquilegia buergeriana* var. *buergeriana*. In this species, geographic variation in spur length has previously been observed in six populations in Utsukushigahara and Norikura regions, but the relationship between spur length and flower visitors in these populations is not known (Hattori et al., 2014). Yellow‐flowered individuals are dominant in this species, and bumblebees seem to be the main flower visitors. In some populations, red‐flowered individuals occur orthotopically with yellow‐flowered individuals, but the genetic relationship between red‐ and yellow‐flowered individuals is unknown. Differences in flower color in *Aquilegia* can lead to genetic isolation even between neighboring or sympatric populations and is likely to be important in speciation (Hopkins & Rausher, 2012; Schemske & Bradshaw, 1999). Our study system is the best one to describe the early genetic isolation of the plant because there is variation not only in spur length but also in flower color.

Here, we first investigated the correspondence between variation in floral spur length and flower‐visiting insect size in 16 *Aquilegia* populations in four mountain regions. The results showed a morphological correlation between spur length and average visitor size in each population, even within the same mountain region; spur lengths were shorter in populations visited by smaller flower visitors, and spur lengths were longer in populations visited by larger flower visitors. Next, we identified genome‐wide single‐nucleotide polymorphisms (SNPs) by the MIG‐seq (multiplexed inter‐simple sequence repeat genotyping by sequencing) method (Suyama & Matsuki, 2015) to clarify the genetic relationships among the populations. These results showed that genetic relationships tended to be clustered by mountain region and, therefore, that spur length evolved in parallel in each mountain region. Individuals with different flower colors were not differentiated in the genome‐wide SNPs analysis, however. This result suggests that pollinator isolation by flower color has not occurred in these populations. Instead, the red flower color is maintained in various populations in which most individuals have yellow flowers.

## 2 MATERIALS AND METHODS

### 2.1 Plant species and study site

**FIGURE 1:**
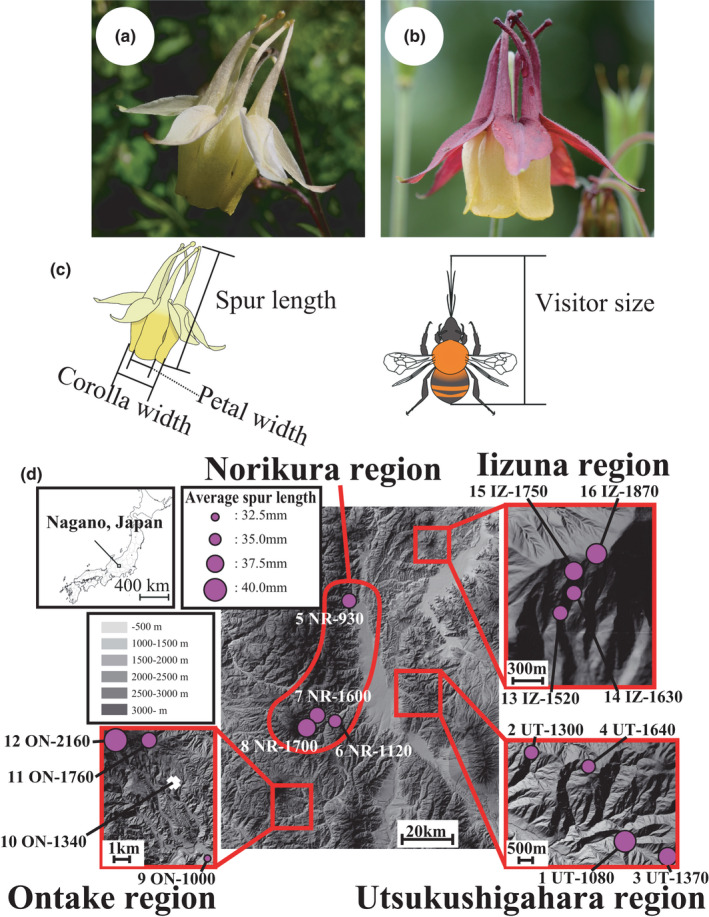
Study species and the study sites. (a) Yellow flower and (b) red flower of *Aquilegia buergeriana* var. *buergeriana*. (c) Measurement of floral spur length and bumblebee size. (d) Locations of the 16 surveyed populations in the four mountain regions (populations are indicated by "Population no. Region abbreviation‐altitude [in meters]"). The size of the purple circle at each site indicates the average spur length of the flowers at that site (spur length was not observed in the 10 ON‐1340 population)

*Aquilegia buergeriana* var. *buergeriana* f. *flavescens* is a perennial, protandrous herb that grows along forest edges in mountain area, endemic to Japan except for Okinawa (Hayashi, 2009). The spur and sepals of its flowers are pale yellow (yellow‐flowered individual) or reddish brown (red‐flowered individuals) (Figure 1a,b). Flowers of both colors face downward. In the study area, in the central Japanese Alps, yellow‐flowered individuals are more common. Japanese *Aquilegia* species are mainly visited by bumblebees (Hattori et al., 2014; Itagaki & Sakai, 2006; Tamura & Shimizu, 1999), even though, in general, yellow‐flowered *Aquilegia* are pollinated by hawkmoths (Hodges et al., 2004). Unlike most *Aquilegia* with yellow flowers, however, the yellow flowers of Japanese *A. buergeriana* do not have protruding anthers and pistils and are not visited by hawkmoths (Toji's personal observation by camera trap for about 3 days in UT‐1640 population).

We studied *Aquilegia* populations in four mountain regions (Utsukushigahara, Norikura, Ontake, and Iizuna) of the central Japanese Alps (Figure 1d; Table S1). Field surveys were conducted during the flowering season, from July to September, in 2018 and 2019: Populations in the Utsukushigahara, Norikura, and Ontake mountain regions were surveyed in 2018, and populations in the Iizuna mountain region were surveyed in 2019.

### 2.2 Measurement of traits

Spur length was measured of all flowering individuals in each population, including both red‐ and yellow‐flowered individuals. The spur lengths of 1–3 randomly selected flowers per plant were measured with a digital caliper (precision 0.01 mm), and the average length of the measured spurs was used as the spur length of that individual (Figure 1c). Corolla diameter and petal width of each individual were measured at the same time. The variation in floral traits was visualized by principal component analysis (PCA) and compared among populations. Preliminary observations showed that the three floral traits did not differ among flowers within an individual, and petal width did not differ among the five petals of each flower. We considered spur length to be the most important trait because of its relation to visitor size. Therefore, in subsequent analyses we focused on spur length. Since the normality of the data was not supported in some populations, we performed a non‐parametric multiple‐comparison Steel–Dwass test to compare average spur length between populations.

We also examined spatial autocorrelation (i.e., whether the variation in spur length could be explained by physical distance) by using the "moran.test" function in the "spdep" package in the R Software Environment ver. 4.0.2 (R Core Team, 2013) to run Moran's I test. This analysis used the average spur length and the latitude and longitude of each population.

### 2.3 Flower visitor assemblages and size variation

To investigate the flower visitors of *A. buergeriana* var. *buergeriana*, we walked through each population and captured insects that were visiting flowers. This survey was conducted during 7:00 a.m. to 2:00 p.m. local time, when flower visitors are active. Each population was observed a total of 60–180 min over 1–3 days at the peak of the flowering season. Captured insects were measured from the tip of the proboscis to the end of the abdomen with a digital caliper (precision, 0.01 mm) to determine visitor size (Figure 1c). Flower visitors tended to simply visit neighboring flowers regardless of the flower color. From this, there may be no differences in visitor assemblage or visiting frequency between differently colored flowers and flowers of the same color.

As the average visitor size for each plant population, the weighted arithmetic average was calculated from the relative abundance of each visitor species and the size of that species:

$$\text{Average}\;\text{visitor}\;\text{size}=\sum_{i=1}^{n}P_{i}\left(N_{i}/N_{t}\right)$$

where *n*, the total number of insect species visiting a *A. buergeriana* var. *buergeriana* population, *Pi*, the average size of the *i*th insect species, *Ni*, the number of flowers in the population that the *i*th insect species visited, and *Nt*, the number of flowers in the population that any of the insect species visited. Thus, *Ni*/*Nt* is the relative abundance of the *i*th insect species visiting the population. In this calculation, the queen and worker of the bumblebees were treated as different insect species. Observations of flower visitor frequency showed that large bumblebees (*Bombus* spp.) were the main visitors, although small bees (*Ceratina* spp.) also visited occasionally. Bumblebees, which extend their proboscis to the nectar source at the tip of the spur to suck nectar, visited both male and female phases and both yellow and red flowers (Toji personal observation). Smaller bees could not reach the spur tip to forage for nectar. Furthermore, although they sometimes collected pollen from the flowers, it is unlikely that they contribute to pollination because they rarely moved between plant individuals and did not visit female phase flowers. Because the pollen visitation patterns of the bumblebees and small bee species were very different, we calculated average visitor size for all visitors (i.e., bumblebees plus small bees) and for bumblebees only.

### 2.4 Factors influencing local spur length

The factors affecting spur length in *A. buergeriana* var. *buergeriana* were estimated by a linear mixed‐model (LMM) analysis. In this analysis, spur length of each individual was used as the objective variable, and average visitor size (all visitors) of each population, average visitor size (only bumblebees) of each population, plant height of each individual, number of flowers per individual, and altitude of each population were used as explanatory variables. Here, plant height and the number of flowers per individual were used as indicators of the nutritional status of the plant, and altitude was used as a representative indicator of non‐biotic environmental factors (e.g., meteorological changes). Population was added to the model as a random effect.

Before conducting the LMM analysis, the variance inflation factor (VIF) statistic was calculated to check for correlation (multicollinearity) between variables, using VIF = 0.5 as the threshold (Neter et al., 1996). For all variables, VIF was less than 0.25, confirming that no multicollinearity existed. Next, we conducted a likelihood ratio test using the parametric bootstrap method (Hoel et al., 1971) to select the effective variables. In this test, for each variable, the difference in deviance, obtained by 1000 bootstrap calculations, between the global model with all variables and a model lacking that variable was determined. No variables were removed as a result of this analysis.

For appropriate model selection, we first prepared a global model that included all of the following variables: average spur length of the population, average visitor size (all visitors) of the population, average visitor size (only bumblebees) of the population, plant height, number of flowers per individual, and altitude. Then, using the "dredge" function of the R package "MuMIn," we compared the global model to simple models with fewer explanatory variables. Then, we adopted the model with the lowest Akaike information criterion (AIC). We excluded the nine ON‐1000 population, which was not visited by bumblebees, from this analysis, treating it as a missing value.

### 2.5 Genetic structure of *A. buergeriana* var. *buergeriana* populations

Leaf samples were obtained from 6–21 individuals in each study population (a total of 232), and DNA was extracted by the CTAB method (Doyle & Doyle, 1990). We performed MIG‐seq (Suyama & Matsuki, 2015) to detect genome‐wide SNPs. A MIG‐seq library was prepared following the protocol of Suyama et al. (2022). A first PCR was performed to amplify inter‐simple sequence repeat regions using MIG‐seq primer set 1 (Suyama & Matsuki, 2015), and then a second PCR was performed on the purified/equalized first PCR product to add the index sequences necessary for sequencing on the MiSeq system and for sample identification. The second PCR products were pooled and fragments of 350 bp or more were isolated. We used MiSeq Reagent Kit v3 (150 cycle, Illumina) and performed sequencing with an Illumina MiSeq Sequencer (Illumina, San Diego, CA, USA) following the manufacturer's protocol. We used the "DarkCycle" option to skip sequencing of the first 17 bases of reads 1 and 2 (simple sequence repeat primer regions and anchors).

Low‐quality reads and extremely short reads containing adaptor sequences were removed by using trimmomatic 0.39 (Bolger et al., 2014). De novo SNP discovery was performed by using the Stacks 2.41 software pipeline (Catchen et al., 2013; Rochette et al., 2019). For de novo SNP discovery, we used the following parameters: minimum depth of coverage required to create a stack (m) = 3, maximum distance between stacks (M) = 2, and maximum mismatches between loci when building the catalog (n) = 2. Three different filtering criteria were applied for quality control of the SNP data. First, SNPs that were retained by 80% or more samples were included in the SNP dataset. Second, SNPs with a minor allele frequency of less than 0.05 were removed. Third, loci containing SNPs with extremely high observed heterozygosity (*H*o ≥ 0.6) were removed. Fourth, after performing a Hardy–Weinberg equilibrium test on each population, we excluded loci where allele frequencies deviated from the Hardy–Weinberg equilibrium at *p* < .01 in three or more populations.

The following population genetic statistics of SNP sites for each population were calculated with the Stacks populations module: expected heterozygosity *H*e, observed heterozygosity *H*o, nucleotide diversity π, and inbreeding coefficient *F*_IS_ (Hartl & Clark, 1997). The population genetic structure was examined by PCA using PLINK 1.9 (Purcell et al., 2007). In addition, a Bayesian clustering analysis was performed with STRUCTURE software version 2.3.4 (Falush et al., 2003; Pritchard et al., 2000). For the STRUCTURE analysis, simulations were performed with 100k burn‐in iterations and 100k Markov chain Monte Carlo iterations. The number of genetic clusters (*K*) was calculated 10 times for each possible *K* value from 1 to 10, and the appropriate number of clusters was estimated based on the Δ*K* value (Evanno et al., 2005). Then, to examine the genetic structure within each mountain region in more detail, we performed additional STRUCTURE analyses. First, SNP re‐detection was performed in each of three mountain regions, the Utsukushigahara, Norikura+Ontake, and Iizuna regions, based on results of the initial analysis. The population structure was obtained based on all samples, with the above filtering criteria used in SNP detection. Second, 10 independent STRUCTURE analysis runs were performed for each mountain region with 100,000 burn‐in steps and an additional 100,000 steps with the admixture model; log‐likelihood values were estimated for each possible *K* value (*K* = 1–10), and the appropriate number of clusters was estimated based on the Δ*K*.

### 2.6 Isolation by distance and isolation by phenotype

We investigated whether the genetic structure of *A. buergeriana* var. *buergeriana* reflects geographic distance or spur length differences. In general, populations separated by greater distances are more genetically differentiated than populations close together (Wright, 1943). On the other hand, if populations with similar traits are also genetically similar, then we can expect to find a correlation between differences in traits between populations and the degree of genetic differentiation. We used GenoDive software version 3.0 (Meirmans, 2020) to calculate the genetic isolation (*F*_ST_) between populations. The geographic distance between populations was calculated from the latitude and longitude of the populations, and the difference in the average spur length of each population was used as the trait difference. We calculated the relationship between pairwise *F*_ST_ or *F*_ST_/(1 – *F*_ST_) and geographic distance between populations, as well as the relationship between pairwise *F*_ST_ or *F*_ST_/(1 – *F*_ST_) and trait difference between populations, following methods in Rousset (1997) and Noutsos et al. (2014). The relationship between genetic isolation and geographic or trait distance was tested by Mantel tests using the R package "ade4" with 10,000 Monte Carlo permutations.

## 3 RESULTS

### 3.1 Spur length and flower visitor size

The average spur length of each population of *A. buergeriana* var. *buergeriana* varied in the range 32.85–40.31 mm, confirming the presence of diversity in spur length within this species (Figure 1d; Figure S1, Table S2). No spatial autocorrelation of average spur length among populations was detected (Moran's I statistic = –0.160, *p* = .757). PCA results for spur length, corolla diameter, and petal width roughly indicated a morphological separation among populations (Figure S2). There appeared to be a morphological separation between the four populations of Utsukushigahara, three populations of Ontake, and the upper and lower elevations of Iizuna. But yellow‐ and red‐flowered individuals could not be clearly separated on the basis of variations in flower morphology (Figure S2).

The average visitor size of each population varied in the range 8.69–40.80 mm (bumblebees plus small bees) and 31.84–40.80 mm (only bumblebees) (Table S1). Five types of bumblebees were observed, in descending order of size: *B. consobrinus* queen, *B. diversus* queen, *B. consobrinus* worker, *B. diversus* worker, and *B. honshuensis* worker. Flower visits by small bees of the genus *Ceratina* were observed in several populations (Table S1). Average plant height of each population varied in the range 54.84–98.01 cm.

### 3.2 Factors influencing local spur length

**FIGURE 2:**
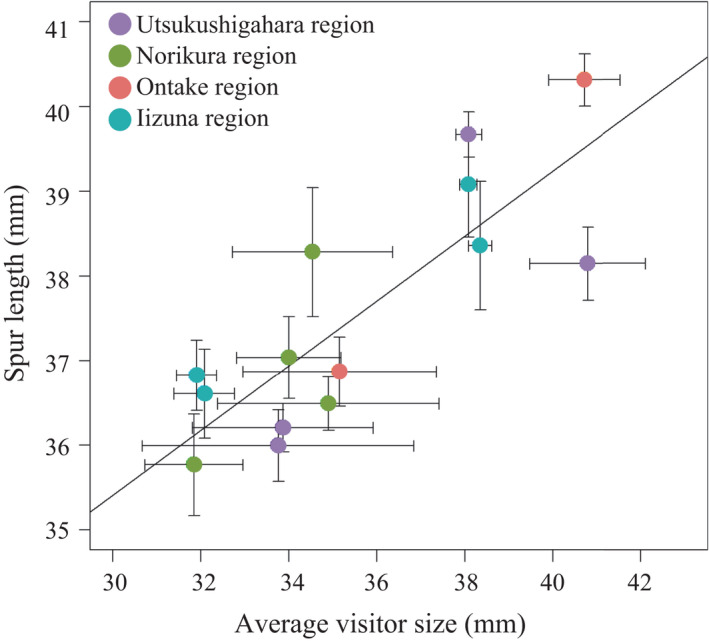
Relationship between average spur length and average visitor size (only bumblebees’ size) in populations of *Aquilegia buergeriana* var. *buergeriana*. A regression line was fitted to the data with reference to the LMM results (*p* < .0001). The 12 ON‐1000 population was not visited by bumblebees, so it was excluded from the analysis. Error bars indicate standard errors

The LMM model with the lowest AIC value was the model that included only average visitor size (only bumblebees) as an explanatory variable (Table 1; Table S3). A very strong linear relationship was found between average spur length and average visitor size (only bumblebees) (*p* < .0001, Figure 2).

**TABLE 1:** The GLM model that best explained variation in average spur length among populations of *Aquilegia buergeriana* var. *buergeriana*

	Coefficient	SE	*t*	*p*‐value
Intercept	23.662	3.159	7.489	<.0001
Average visitor size (only bumblebees)	0.388	0.088	4.420	<.0001

Note

This model had the lowest AIC value among the tested models; see Table S2 for the model comparison results.

### 3.3 Genetic structure of *A. buergeriana* var. *buergeriana* populations

A total of 16,033,406 raw reads (69,109 ± 731 reads per sample) were obtained by MIG‐seq, and after quality control, 15,510,825 reads (66,587 ± 713 reads per sample) were used for further analyses. After de novo SNP detection and filtering, the MIG‐seq dataset of 232 samples from 16 populations contained 190 SNPs, distributed among the mountain regions as follows: Utsukushigahara region (63 individuals, 175 SNPs), Norikura+Ontake region (126 individuals, 167 SNPs), and Iizuna region (43 individuals, 175 SNPs). Norikura and Ontake regions were combined based on the initial STRUCTURE results. The values of the population genetics parameters varied among populations (*H*e, 0.0904–0.1450; *H*o, 0.0593–0.2154; π, 0.0616–0.2248; *F*_IS_, –0.0608 to 0.2041; Table S4).

**FIGURE 3:**
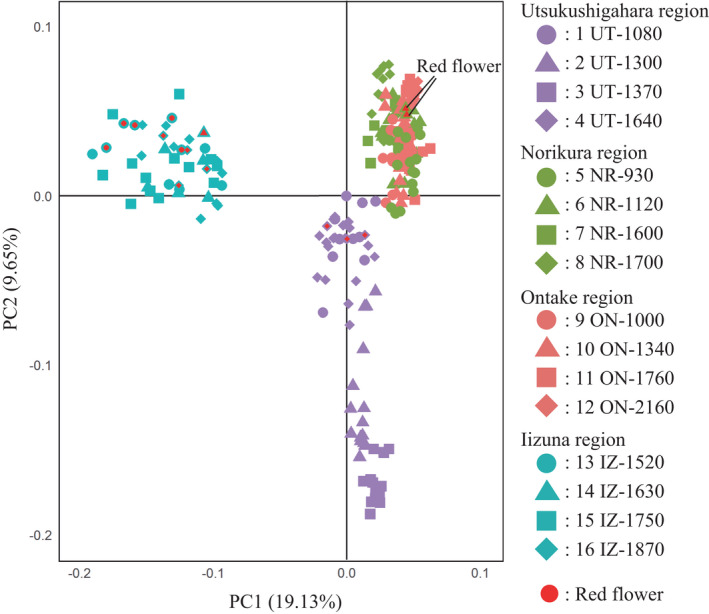
Principal component analysis results for all populations based on 190 SNPs data. Principal component 1 (PC1; contribution rate 19.13%) is plotted on the horizontal axis and PC2 (contribution rate 9.65%) on the vertical axis. The red‐flowered individuals sampled in some populations are shown by symbols with a red center

**FIGURE 4:**
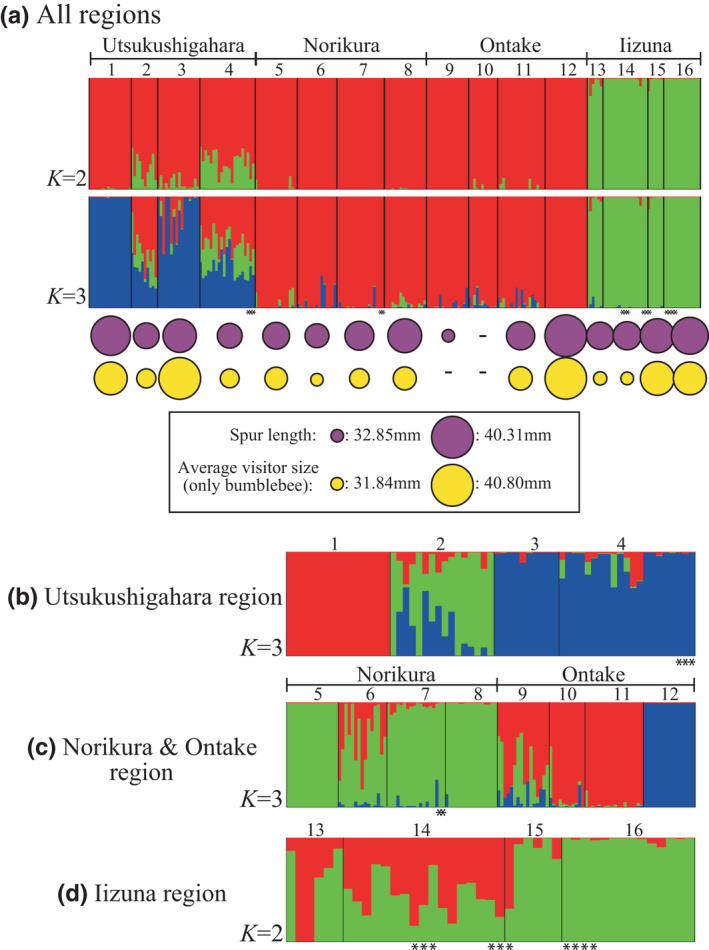
Bayesian genetic clustering analysis STRUCTURE results based on SNPs data. In each case, the appropriate value of *K* was determined from Δ*K* (see Figure S3). Population numbers, corresponding to those in Figure 1, are shown along the top of each panel. Populations in which red‐flowered individuals were sampled are indicated by asterisks along the bottom of each panel. (a) Cluster analysis results for SNPs in all populations (*K* = 2, 3). The relative relationship between average spur length relative and average visitor size (only bumblebees) in each population is shown by the relative sizes of the purple and yellow circles. Cluster analysis results for SNPs of only the (b) Utsukushigahara (*K* = 3), (c) Norikura + Ontake (*K* = 3), and (d) Iizuna (*K* = 2) regions

In the PCA results for 190 SNPs of 232 individuals from 16 populations of *A. buergeriana* var. *buergeriana*, principal components 1 and 2 (PC1 and PC2, respectively) explained 28.78% of the variance. The geographical structure of the populations is clearly reflected in a plot of PC2 against PC1 (Figure 3), but within populations of *A. buergeriana var. buergeriana*, yellow‐ and red‐flowered individuals did not clearly show genetic isolation. On the basis of the PCA results, the populations could be separated into three regional groups: Utusuhigahara, Norikura+Ontake, and Iizuna populations. The STRUCTURE analysis of all populations showed that, based on Δ*K*, the appropriate number of genetic clusters was *K* = 2 (most likely) or *K* = 3 (next most likely) (Figure S3). The STRUCTURE analysis results also clearly reflected the geographical structure in each region (Figure 4; Figures S3 and S4). The appropriate number of genetic clusters in the Utsukushigahara (63 individuals, 175 SNPs), Ontake+Norikura (126 individuals, 167 SNPs), and Iizuna (43 individuals, 175 SNPs) mountain regions were *K* = 3, 3, and 2, respectively, based on Δ*K* (Figure S3). Structure among populations within the same mountain region was also detected (Figure 4b–d). In particular, the populations in Norikura+Ontake region could be separated into Norikura and Ontake groups. These two groups were not separated in the initial STRUCTURE analysis. Yellow‐ and red‐flowered individuals in a population were not genetically distinguished in the STRUCTURE analysis results.

### 3.4 Isolation by distance and isolation by phenotype

A significant relationship between geographic distance and genetic isolation (*F*_ST_, *F*_ST_/(1 − *F*_ST_)) was detected (Table 2). On the other hand, differences in average spur length between populations were not related to genetic isolation.

**TABLE 2:** Mantel test results for the relationships between *F*_ST_ or *F*_ST_/(1 − *F*_ST_) and geographic distance or spur length differences

	*F*_ST_	*F*_ST_/(1 − *F*_ST_)
Geographic distance	**<.0001**	**<.0001**
log(Geographic distance)	**<.0001**	.**0032**
Spur length differences	.9748	.9545

Note

For each test, statistically significant *p*‐values are shown in bold.

## 4 DISCUSSION

### 4.1 Intraspecific independent evolution of spur length among mountain regions

The spur length of *A. buergeriana* var. *buergeriana* was correlated with the average flower visitor size (only bumblebees) of the population; spur length varied with the average visitor size even among spatially close populations in the same mountain region (Figures 2 and 4a). The genetic results obtained by PCA and the STRUCTURE analysis of genome‐wide SNPs suggest that populations within each mountain region are more closely related to each other than to populations in other mountain regions (Figures 3 and 4; Figure S4). Genetic isolation was proportional to geographical distance and did not reflect spur length differences (Table 2). These results suggest that genetic origin is derived from each mountain region, and spur length of *A. buergeriana* var. *buergeriana* evolved independently in each mountain region.

In the Iizuna region, populations at different altitudes seem to belong to different genetic clusters (Figure 4d); furthermore, flowers in lower‐altitude populations were visited by *B. diversus* and those in higher‐altitude populations were visited by *B. consobrinus* (Table S1). These results suggest that genetic differentiation may occur between higher‐ and lower‐altitude populations because of a lack of pollinator sharing. Further studies are needed to determine whether gene flow by pollination is hindered between populations at higher and lower altitudes in the Iizuna region.

In anole lizards, leg length has evolved independently on different islands to suit local habitats (Losos, 2010), and in stickleback fishes, the evolution of marine to freshwater forms (sticklebacks that move between rivers and the sea) occurred independently in different marine and freshwater locations in various regions of the world (Jones et al., 2012). We propose that plant species distributed across a wide geographic range with site‐specific, different‐sized pollinators constitute another model suitable for testing independent adaptive radiation. We have demonstrated that spur length in an *Aquilegia* species may have evolved independently among mountain regions by using a population genetics approach to compare traits among mountain regions. Independent evolution in different mountain regions has recently been examined in various model systems: for example, the independent evolution of upland and short‐winged forms of scorpionfly *Panorpodes* (Panorpodidae) (Suzuki et al., 2019), the independent evolution of *Potentilla matsumurae* (Rosaceae) in fellfield and snowbed environments on different mountains in Japan (Hirao et al., 2019), and the independent evolution of alpine morphology in *Antirrhinum* species (Antirrhineae) (Durán‐Castillo et al., 2021). Furthermore, we recently presented a case in which we used microsatellite markers to show the independent adaptation of floral tube size in *Lamium album* var. *barbatum* (Lamiaceae), associated with flower visitor size, in the Utsukushigahara and Norikura regions of the Japanese Alps (Toji et al., 2021). These examples show that comparisons between mountain regions can be used to study independent trait evolution in various organisms, and similar patterns might be found in many places around the world.

### 4.2 Flower color does not contribute to genetic isolation

Although red‐flowered individuals were observed in some populations, genetic analyses (STRUCTURE and PCA) based on neutral genes did not differentiate red‐ and yellow‐flowered individuals in those populations. These results suggest that red flower color is maintained in each population merely as a flower color polymorphism. Although subtle plasticity of floral color has been reported in *A. coerulea*, color changes have not occurred so much as the *A. buergeriana* var. *buergeriana* (Brunet & Van Etten, 2019). Throughout the diversification history of *Aquilegia*, flower color changes have been shown to be associated with pollinator shifts (Whittall & Hodges, 2007). Another well‐known example is the *Mimulus aurantiacus* species complex, in which flower color influences pollinator preference, which in turn leads to genetic isolation. Within the *M. aurantiacus* species complex, there are two ecotypes, one with red flowers, which are preferred by hummingbirds, and the other with yellow flowers, which are preferred by hawkmoths. Although these two ecotypes are very closely related, cluster analysis by RAD‐seq (restriction site‐associated DNA sequencing) based on genome‐wide SNP data has shown that they are genetically distinct (Sobel & Streisfeld, 2015). In the hybrid zone between the two ecotypes, the *MaMyb2* gene, which is involved in the synthesis of flower pigments, is geographically maintained despite neutral gene flow occurred (Sobel & Streisfeld, 2015; Stankowski & Streisfeld, 2015; Streisfeld & Kohn, 2005). Gene flow between yellow and red flower *M. aurantiacus* ecotypes in the early stages of speciation seems to be limited mainly by differences in pollinator preference (Sobel & Streisfeld, 2015). Similarly, gene flow between two closely related *Aquilegia* species: hummingbird‐pollinated, red‐flowered *A. formosa* and hawkmoth‐pollinated, yellow‐flowered *A. pubescens* are also limited by pollinator isolation when the two species are distributed parapatrically (Fulton & Hodges, 1999; Noutsos et al., 2014).

Why yellow‐ and red‐flowered individuals in *A. buergeriana* var. *buergeriana* may not become genetically isolated? In the central Nagano region, where this study was conducted, bumblebees appear to be abundant and many flowers depend on bumblebees for pollination (e.g., Egawa & Itino, 2020), whereas potential pollinators such as birds and butterflies that prefer red flowers are scarce. In another Japanese mountain region (the Taisetsu mountains), flowers are dominantly visited by bees and flies at the community level (Mizunaga & Kudo, 2017). A recent review has reported that Lepidoptera account for less than 10% of insect visitors to flowers in many parts of Asia, whereas bees and flies account for more than half (Funamoto, 2019). It is possible that in the central Japanese Alps, because only the locally abundant bumblebees contribute to pollination of *A. buergeriana* var. *buergeriana* irrespective of the flower color, pollinator shifts to other taxa such as birds have not triggered the evolution of extreme traits. Whether bumblebees cause selection or act neutrally with respect to flower color requires further investigation, but the maintenance of small numbers of red‐flowered individuals in some populations suggests that the frequency of red flowers may be determined by genetic drift. The maintenance of this small number of different flower color polymorphisms in some populations might become a driving force for a pollinator shift should the plants be faced with a new pollinator environment. Although it should be noted that the number of red‐flowered individuals was very low (15 samples) throughout the study, it is an important finding to consider the relationship between flower color and pollinator in *Aquilegia*.

## 5 CONCLUSIONS

Two main conclusions follow from our results that (1) the evolution of spur length in *A. buergeriana* var. *buergeriana* has occurred independently in different mountain regions, and (2) the few red‐flowered phenotypes that occur within the species may not lead to genetic differentiation. First, given that the independent evolution of floral size in different mountain regions has also recently been reported in *L. album* var. *barbatum* (Toji et al., 2021), the independent evolution of floral size among mountain regions may be a generalized event that occurs commonly in different taxa. The approach used here to test for independent evolution among mountain regions is applicable to any taxon and a variety of traits. In particular, morphological analyses combined with MIG‐seq (Suyama & Matsuki, 2015), which can be used to obtain genome‐wide SNPs from non‐model organisms, constitute a powerful method for elucidating patterns of morphological and genetic diversification within species. Second, we found no relationship between flower color and the degree of genetic differentiation, despite the fact that pollinator isolation caused by differences in flower color has been reported in two closely related species of *Aquilegia* (Fulton & Hodges, 1999; Noutsos et al., 2014). We infer that in the mountainous region of Japan, where bumblebees are locally abundant large pollinators, shifts to different pollinator taxa are unlikely to occur, and the polymorphism in *A. buergeriana* var. *buergeriana* flower color is likely maintained by random genetic drift. Thus, our results are an important exception to diversification in genus *Aquilegia*, which is well known to have occurred by both flower color and pollinator shifts (Whittall & Hodges, 2007).

## CONFLICT OF INTEREST

There are no conflicts of interest.

## AUTHOR CONTRIBUTIONS

**Tsubasa Toji:** Conceptualization (equal); Data curation (equal); Formal analysis (equal); Funding acquisition (equal); Investigation (equal); Methodology (equal); Visualization (equal); Writing – original draft (equal). **Shun K Hirota:** Formal analysis (lead); Methodology (lead); Software (lead). **Natsumi Ishimoto:** Data curation (lead); Investigation (lead). **Yoshihisa Suyama:** Formal analysis (supporting); Supervision (lead); Writing – review & editing (lead). **Takao Itino:** Conceptualization (lead); Funding acquisition (equal); Project administration (lead); Writing – original draft (lead); Writing – review & editing (lead).

## Supporting information

Supplementary Material

## Data Availability

All raw MIG‐seq data have been deposited in the DDBJ Sequence Read Archive (DRA) with accession number DRA012638. Field data have been made open access and deposited onto Dryad: https://doi.org/10.5061/dryad.4tmpg4fb9.
