# Supplementary material for: Intraspecific independent evolution of floral spur length in response to local flower visitor size in Japanese Aquilegia in different mountain regions
Source: Ecol Evol. 2022 Mar 1;12(3):e8668. doi: 10.1002/ece3.8668 (PMC8888250; doi:10.1002/ece3.8668)
Supplement: Supplementary file 1 — Supplementary Material [file ECE3-12-e8668-s001.docx]

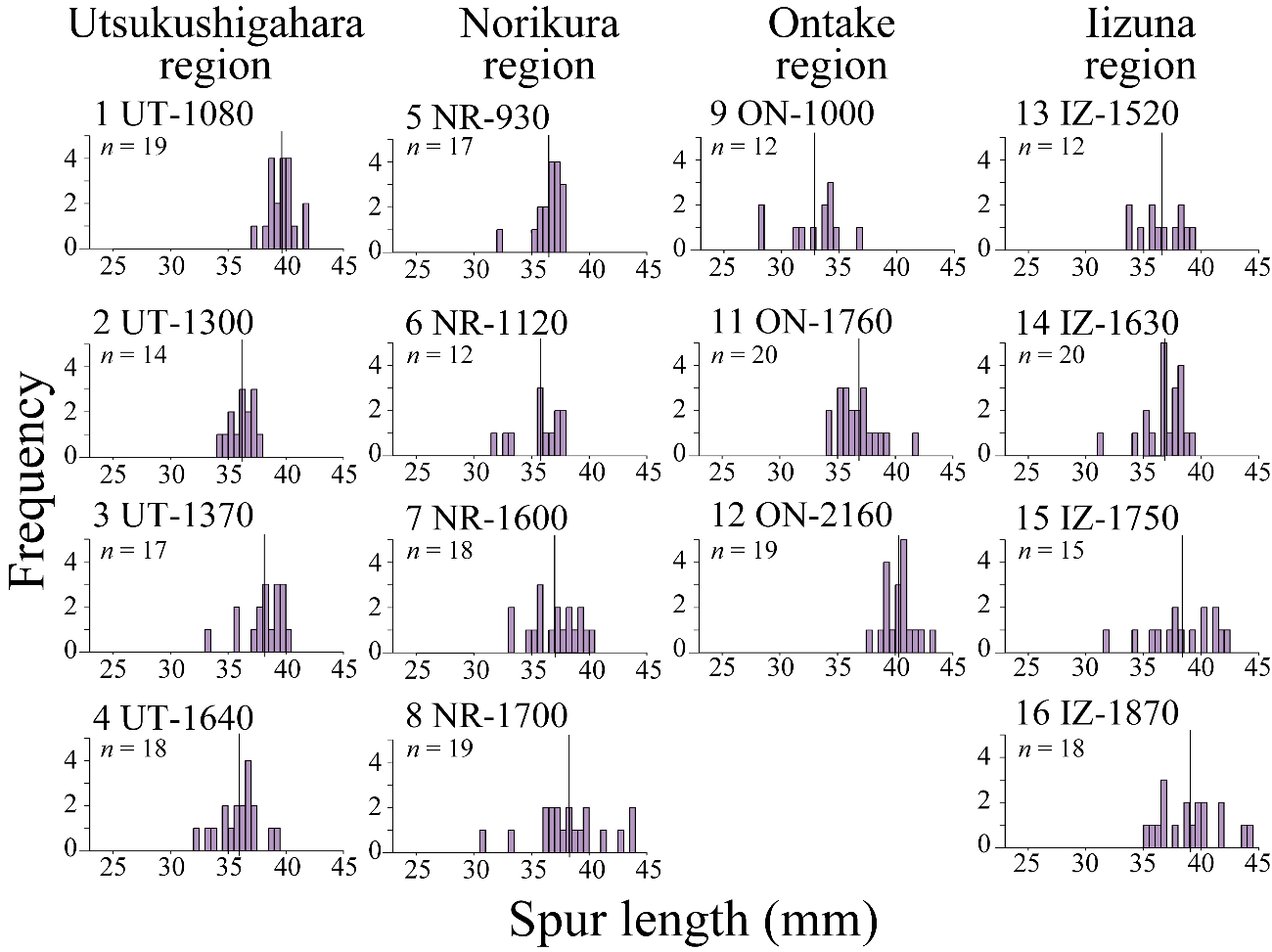


Figure S1. Frequency distributions of spur length in populations of *Aquilegia buergeriana var. buergeriana*. The vertical line in each histogram indicates the mean spur length in that population. *n* indicates the sample size. Spur length was not measured in the 10 ON-1340 population.


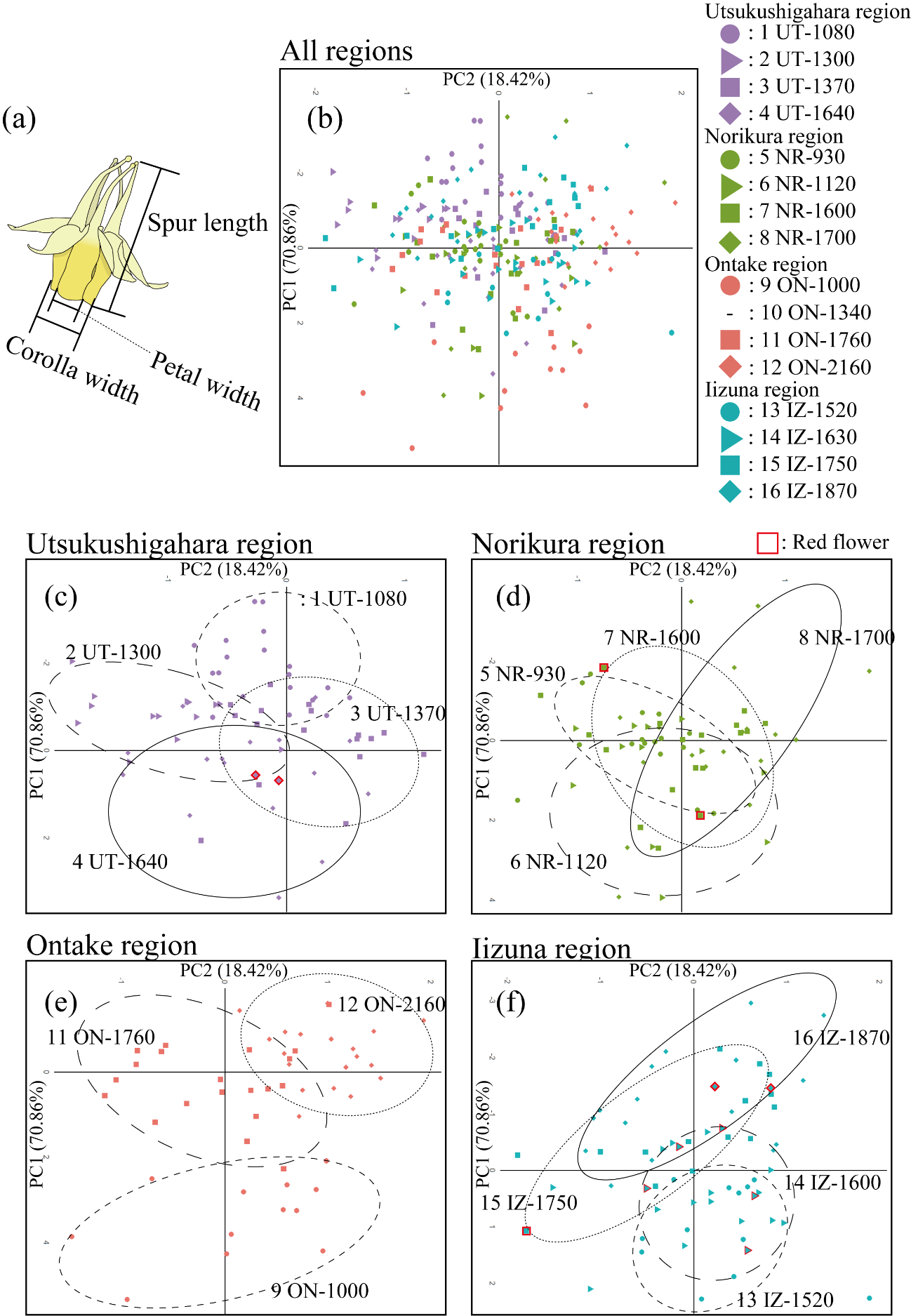


Figure S2. Principal component analysis (PCA) results for three floral traits in *A. buergeriana var. buergeriana*. (a) Measurement of each trait. (b) PCA results for all floral traits of individuals in all populations. (c–f) PCA results for all floral traits of individuals in the populations of each mountain region. Ellipses indicate the different population groupings. Symbols for red-flowered individuals are outlined in red.


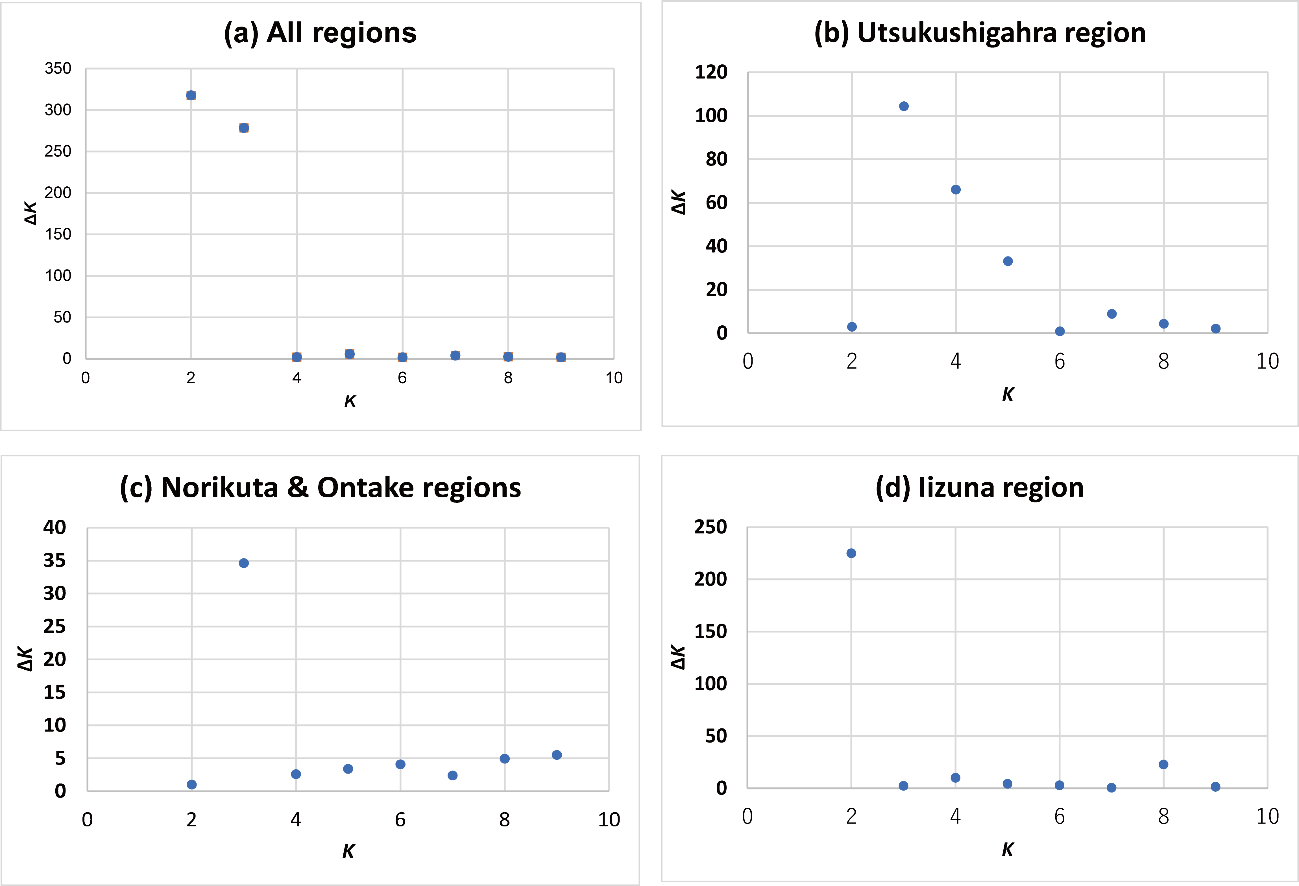


Figure S3. Number of appropriate clusters (*K*) determined by the STRUCTURE analysis based on Δ*K*. (a) When all populations were included in the analysis, *K* = 2 or *K* = 3 was inferred to be the appropriate number of clusters. (b) For populations in the Utsukushigahara region only, *K* = 3 was appropriate. (c) For Norikura+Ontake populations only, *K* = 3 was appropriate. (d) For Iizuna populations only, *K* = 2 was appropriate.


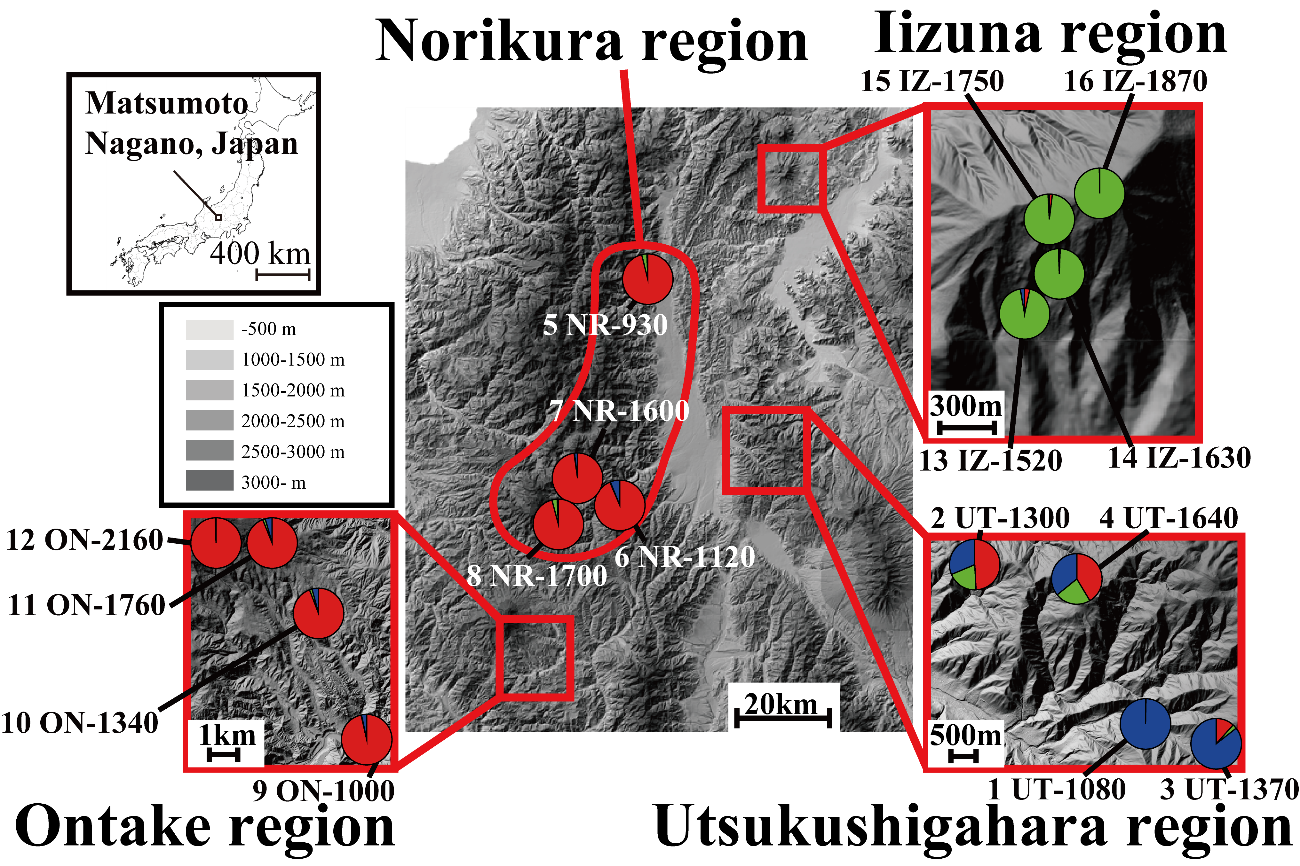


Figure S4. The results of the STRUCTURE analysis for all populations with K = 3. For each population, the circle graph shows that the relative probability of the population belonging to each of the four clusters (indicated by different colors). To obtain the probability of the population belonging to a cluster, the probabilities that the individuals in the population belonged to the cluster were averaged.

Table S1. Overview of the study site. Location information and numbers of flower visiting insects observed (W, worker; Q, Queen).

|  |  |  |  |  | Observed flower visitors | | | | | |  |
| --- | --- | --- | --- | --- | --- | --- | --- | --- | --- | --- | --- |
| Pop No. | Population name | Mountain region | Latitude | Longitude | Small bees | *Bombus honshuensis* W | *B. diversus* W | *B. diversus* Q | *B. consobrinus* W | *B. consobrinus* Q | Observation time (min) |
| 1 | UT-1080 | Utsukushigahara | 36.222378 | 138.070700 | - | - | - | - | 5 | - | 180 |
| 2 | UT-1300 | Utsukushigahara | 36.250953 | 138.034308 | - | - | 3 | 1 | - | - | 150 |
| 3 | UT-1370 | Utsukushigahara | 36.215731 | 138.088757 | - | - | - | - | 4 | 1 | 100 |
| 4 | UT-1640 | Utsukushigahara | 36.246782 | 138.055615 | 3 | 2 | - | - | 4 | - | 120 |
| 5 | NR-930 | Norikura | 36.539493 | 137.785174 | - | - | 2 | 1 | - | - | 140 |
| 6 | NR-1120 | Norikura | 36.129036 | 137.719438 | 2 | - | 2 | - | - | - | 60 |
| 7 | NR-1600 | Norikura | 36.144650 | 137.628887 | - | 4 | - | - | 4 | - | 180 |
| 8 | NR-1700 | Norikura | 36.109987 | 137.607450 | 9 | - | 4 | 2 | - | - | 100 |
| 9 | ON-1000 | Ontake | 35.800620 | 137.564363 | 1 | - | - | - | - | - | 100 |
| 10 | ON-1340 | Ontake | 35.842808 | 137.541307 | - | - | - | - | - | - | - |
| 11 | ON-1760 | Ontake | 35.866656 | 137.525053 | 2 | - | 2 | - | 2 | - | 155 |
| 12 | ON-2160 | Ontake | 35.869647 | 137.500463 | - | - | - | - | 3 | - | 150 |
| 13 | IZ-1520 | Iizuna | 36.731352 | 138.125567 | - | - | 4 | - | - | - | 120 |
| 14 | IZ-1630 | Iizuna | 36.733123 | 138.126941 | - | - | 3 | - | - | - | 120 |
| 15 | IZ-1750 | Iizuna | 36.734757 | 138.127005 | - | - | - | - | 3 | - | 120 |
| 16 | IZ-1870 | Iizuna | 36.736391 | 138.129494 | - | - | - | - | 3 | - | 120 |

Table S2. Continued. Average spur length and average plant height in each population. Statistically significant differences between populations are indicated by different lowercase letters (Steel-Dwass test, *P* < 0.05).

|  |  | Average pollinator size (mm) | |  |  |
| --- | --- | --- | --- | --- | --- |
| Pop No. | Population name | All pollinators | Only bumblebees | Average spur length (mm) | Average plant height (cm) |
| 1 | UT-1080 | 38.09 | 38.09 | 39.67 a | 86.05 abcd |
| 2 | UT-1300 | 33.86 | 33.86 | 36.20 c | 81.82 abcd |
| 3 | UT-1370 | 40.80 | 40.80 | 38.15 abc | 98.01 a |
| 4 | UT-1640 | 27.47 | 33.76 | 35.99 c | 76.14 bcd |
| 5 | NR-930 | 34.90 | 34.90 | 36.49 c | 92.41 ab |
| 6 | NR-1120 | 20.30 | 31.84 | 35.77 c | 80.26 abcd |
| 7 | NR-1600 | 34.00 | 34.00 | 37.04 bc | 69.59 de |
| 8 | NR-1700 | 27.81 | 34.54 | 38.28 abc | 72.62 cde |
| 9 | ON-1000 | 8.69 | - | 32.85 d | 66.32 de |
| 10 | ON-1340 | - | - | - | - |
| 11 | ON-1760 | 25.68 | 35.15 | 36.87 bc | 57.51 e |
| 12 | ON-2160 | 32.18 | 40.72 | 40.31 a | 56.56 e |
| 13 | IZ-1520 | 36.61 | 32.08 | 36.61 c | 54.84 e |
| 14 | IZ-1630 | 31.90 | 31.90 | 36.82 c | 58.95 e |
| 15 | IZ-1750 | 38.35 | 38.35 | 38.36 ab | 67.71 de |
| 16 | IZ-1870 | 38.08 | 38.08 | 39.08 ab | 63.67 de |

Table S3. GLM model selection results obtained by using the dredge function in the "MuMIn" package.

| Predictive variables and coefficients | | | | |  |  |  |  |  |  |
| --- | --- | --- | --- | --- | --- | --- | --- | --- | --- | --- |
| Altitude | Plant height | Number of flowers per ramet | Average visitor size (all visitors) | Average visitor size (only bumblebees) | Intercept | Degrees of freedom | Log likelihood | AIC | Delta AIC | Weight |
|  |  |  |  | 0.38760 | 23.66 | 4 | -514.391 | 1036.8 | 0 | 0.895 |
|  |  |  | -0.02337 | 0.41750 | 23.35 | 5 | -516.194 | 1042.4 | 5.61 | 0.054 |
|  |  |  |  |  | 37.57 | 3 | -519.135 | 1044.3 | 7.49 | 0.021 |
|  | 0.00861 |  |  | 0.38480 | 23.14 | 5 | -517.756 | 1045.5 | 8.73 | 0.011 |
|  |  | 0.00003 |  | 0.38760 | 23.66 | 5 | -518.076 | 1046.2 | 9.37 | 0.008 |
|  |  |  | 0.11770 |  | 33.7 | 4 | -519.376 | 1046.8 | 9.97 | 0.006 |
| 0.00087 |  |  |  | 0.35590 | 23.47 | 5 | -520.011 | 1050 | 13.24 | 0.001 |
|  | 0.00930 |  | -0.03049 | 0.42370 | 22.69 | 6 | -519.466 | 1050.9 | 14.15 | 0.001 |
|  |  | 0.00009 | -0.02338 | 0.41750 | 23.35 | 6 | -519.875 | 1051.8 | 14.97 | 0.001 |
|  | 0.01184 |  |  |  | 36.71 | 4 | -522.019 | 1052 | 15.26 | 0 |
|  |  | 0.00127 |  |  | 37.55 | 4 | -522.776 | 1053.6 | 16.77 | 0 |
|  | 0.01131 | -0.00674 |  | 0.38570 | 23 | 6 | -521.131 | 1054.3 | 17.48 | 0 |
|  | 0.01065 |  | 0.11230 |  | 33.1 | 5 | -522.454 | 1054.9 | 18.13 | 0 |
| 0.00188 |  |  |  |  | 34.7 | 4 | -523.65 | 1055.3 | 18.52 | 0 |
| 0.00087 |  |  | 0.00004 | 0.35560 | 23.47 | 6 | -521.819 | 1055.6 | 18.86 | 0 |
|  |  | 0.00113 | 0.11760 |  | 33.69 | 5 | -523.023 | 1056 | 19.27 | 0 |
| 0.00195 |  |  | 0.12220 |  | 30.57 | 5 | -523.562 | 1057.1 | 20.34 | 0 |
| 0.00132 | 0.01277 |  |  | 0.33520 | 22.59 | 6 | -522.743 | 1057.5 | 20.7 | 0 |
| 0.00090 |  | 0.00197 |  | 0.35440 | 23.45 | 6 | -523.662 | 1059.3 | 22.54 | 0 |
|  | 0.01217 | -0.00717 | -0.03302 | 0.42790 | 22.51 | 7 | -522.815 | 1059.6 | 22.85 | 0 |
|  | 0.01451 | -0.00700 |  |  | 36.61 | 5 | -525.369 | 1060.7 | 23.96 | 0 |
| 0.00236 | 0.01571 |  |  |  | 32.82 | 5 | -525.84 | 1061.7 | 24.9 | 0 |
| 0.00134 | 0.01289 |  | 0.00281 | 0.33070 | 22.62 | 7 | -524.533 | 1063.1 | 26.28 | 0 |
|  | 0.01314 | -0.00640 | 0.11140 |  | 33.04 | 6 | -525.834 | 1063.7 | 26.89 | 0 |
| 0.00241 | 0.01495 |  | 0.11570 |  | 29 | 6 | -525.893 | 1063.8 | 27 | 0 |
| 0.00191 |  | 0.00324 |  |  | 34.59 | 5 | -527.248 | 1064.5 | 27.71 | 0 |
| 0.00090 |  | 0.00193 | 0.00097 | 0.35280 | 23.47 | 7 | -525.465 | 1064.9 | 28.15 | 0 |
| 0.00132 | 0.01541 | -0.00666 |  | 0.33600 | 22.46 | 7 | -526.123 | 1066.2 | 29.46 | 0 |
| 0.00199 |  | 0.00370 | 0.12220 |  | 30.46 | 6 | -527.151 | 1066.3 | 29.52 | 0 |
| 0.00236 | 0.01831 | -0.00680 |  |  | 32.72 | 6 | -529.202 | 1070.4 | 33.62 | 0 |
| 0.00133 | 0.01555 | -0.00675 | 0.00010 | 0.33550 | 22.45 | 8 | -527.905 | 1071.8 | 35.03 | 0 |
| 0.00241 | 0.01722 | -0.00588 | 0.11480 |  | 28.95 | 7 | -529.301 | 1072.6 | 35.82 | 0 |

Table S4. Population genetics parameters of each population.

| Pop No. | Population | Number of analyzed individuals | Observed heterozygosity (*H*o) | SE | Expected heterozygosity (*H*e) | SE | Nucleotide diversity (π) | SE | Fixation index (*F*_IS_) | SE |
| --- | --- | --- | --- | --- | --- | --- | --- | --- | --- | --- |
| 1 | UT-1080 | 16 | 0.1347 | 0.0174 | 0.1155 | 0.0139 | 0.1199 | 0.0144 | -0.0339 | 0.1249 |
| 2 | UT-1300 | 10 | 0.1235 | 0.0154 | 0.1437 | 0.0129 | 0.1526 | 0.0138 | 0.0954 | 0.0795 |
| 3 | UT-1370 | 16 | 0.1437 | 0.0137 | 0.1578 | 0.0132 | 0.1637 | 0.0137 | 0.0558 | 0.1236 |
| 4 | UT-1640 | 21 | 0.1450 | 0.0120 | 0.2112 | 0.0136 | 0.2169 | 0.0140 | 0.2041 | 0.1382 |
| 5 | NR-930 | 16 | 0.0786 | 0.0132 | 0.0897 | 0.0117 | 0.0929 | 0.0121 | 0.0360 | 0.0928 |
| 6 | NR-1120 | 15 | 0.1198 | 0.0131 | 0.1531 | 0.0132 | 0.1587 | 0.0137 | 0.1186 | 0.0984 |
| 7 | NR-1600 | 18 | 0.0919 | 0.0114 | 0.1378 | 0.0125 | 0.1422 | 0.0129 | 0.1636 | 0.1225 |
| 8 | NR-1700 | 16 | 0.1254 | 0.0147 | 0.1443 | 0.0137 | 0.1497 | 0.0142 | 0.0799 | 0.1350 |
| 9 | ON-1000 | 16 | 0.0951 | 0.0108 | 0.1581 | 0.0132 | 0.1641 | 0.0137 | 0.1967 | 0.1515 |
| 10 | ON-1340 | 11 | 0.1024 | 0.0106 | 0.1470 | 0.0129 | 0.1549 | 0.0136 | 0.1416 | 0.0895 |
| 11 | ON-1760 | 18 | 0.1447 | 0.0137 | 0.1632 | 0.0137 | 0.1688 | 0.0142 | 0.0549 | 0.1469 |
| 12 | ON-2160 | 16 | 0.0904 | 0.0179 | 0.0593 | 0.0110 | 0.0616 | 0.0114 | -0.0608 | 0.1432 |
| 13 | IZ-1520 | 6 | 0.1352 | 0.0131 | 0.2154 | 0.0152 | 0.2248 | 0.0158 | 0.2018 | 0.1109 |
| 14 | IZ-1630 | 17 | 0.1311 | 0.0144 | 0.2089 | 0.0154 | 0.2213 | 0.0163 | 0.2026 | 0.1100 |
| 15 | IZ-1750 | 6 | 0.1258 | 0.0165 | 0.1843 | 0.0149 | 0.2026 | 0.0163 | 0.1861 | 0.0576 |
| 16 | IZ-1870 | 14 | 0.1244 | 0.0148 | 0.1616 | 0.0147 | 0.1684 | 0.0153 | 0.1092 | 0.1306 |
